# Ncf1 affects osteoclast formation but is not critical for postmenopausal bone loss

**DOI:** 10.1186/s12891-016-1315-1

**Published:** 2016-11-09

**Authors:** Alexandra Stubelius, Annica Andersson, Rikard Holmdahl, Claes Ohlsson, Ulrika Islander, Hans Carlsten

**Affiliations:** 1 Department of Rheumatology and Inflammation Research, Centre for Bone and Arthritis Research (CBAR), Sahlgrenska Academy, University of Gothenburg, Box 480, 405 30 Göteborg, Sweden; 2 Department of Internal Medicine and Clinical nutrition, Centre for Bone and Arthritis Research (CBAR), Sahlgrenska Academy, University of Gothenburg, Gothenburg, Sweden; 3 Medical Inflammation Research, Karolinska Institutet, Stockholm, Sweden

**Keywords:** Reactive Oxygen Species, Neutrophil cytosolic factor 1, Ncf1, Postmenopausal bone loss, NOX 2, Estrogen Deficiency, Bone mineral density, Osteoclasts, Osteoimmunology, Pre-osteoclasts

## Abstract

**Background:**

Increased reactive oxygen species and estrogen deficiency contribute to the pathophysiology of postmenopausal osteoporosis. Reactive oxygen species contribute to bone degradation and is necessary for RANKL-induced osteoclast differentiation. In postmenopausal bone loss, reactive oxygen species can also activate immune cells to further enhance bone resorption. Here, we investigated the role of reactive oxygen species in ovariectomy-induced osteoporosis in mice deficient in Ncf1, a subunit for the NADPH oxidase 2 and a well-known regulator of the immune system.

**Methods:**

B10.Q wild-type (WT) mice and mice with a spontaneous point mutation in the Ncf1-gene (Ncf1*/*) were ovariectomized (ovx) or sham-operated. After 4 weeks, osteoclasts were generated *ex vivo*, and bone mineral density was measured using peripheral quantitative computed tomography. Lymphocyte populations, macrophages, pre-osteoclasts and intracellular reactive oxygen species were analyzed by flow cytometry.

**Results:**

After ovx, Ncf1*/*-mice formed fewer osteoclasts *ex vivo* compared to WT mice. However, trabecular bone mineral density decreased similarly in both genotypes after ovx. Ncf1*/*-mice had a larger population of pre-osteoclasts, whereas lymphocytes were activated to the same extent in both genotypes.

**Conclusion:**

Ncf1*/*-mice develop fewer osteoclasts after ovx than WT mice. However, irrespective of genotype, bone mineral density decreases after ovx, indicating that a compensatory mechanism retains bone degradation after ovx.

## Background

Bone remodeling occurs throughout life, shifting to favor bone resorption in postmenopausal women. Osteoclasts (OCL) resorb bone, and the presence of reactive oxygen species (ROS) at the interface between OCL and bone suggests a role for ROS in bone resorption [1–4]. ROS are produced by membrane-localized nicotinamide adenine dinucleotide phosphate (NADPH) oxidases (NOX); [5], where NOX 1, 2, and 4 are active in OCL and their myeloid precursor cells [1, 4]. The activity of NOX 2 is regulated by the neutrophil cytosolic factor 1 (Ncf1, alias p47^phox^) subunit [6]. ROS from NOX 2 kills bacteria in professional phagocytes (such as macrophages and neutrophils with highly efficient phagocytosis), and damages cells and tissues during inflammation. However, reduced production of ROS in mice lacking Ncf1 promotes T-cell dependent autoimmune diseases [6]. Further, T-cells activated by antigen presenting cells (APCs) induce bone degradation in postmenopausal osteoporosis. After ovariectomy (ovx), the excess tumor necrosis factor alpha (TNFα) from T-cells activates OCL and results in reduced bone density [7–9]. Other mechanisms underlying postmenopausal bone loss include increased receptor activator of nuclear factor κ B ligand (RANKL) signaling [10] and increased OCL formation [11].

To investigate the role of ROS and Ncf1 in postmenopausal bone loss, we employed a postmenopausal bone-loss model by ovx or sham operating mice with a spontaneous mutation in Ncf1. We investigated osteoclastogenesis *ex vivo*, used peripheral quantitative computed tomography (pQCT) for measuring bone mineral density (BMD), and flow cytometry was used to investigate pre-OCL and immune cells, including immune cell activation. We found that bone mineral density decreased to the same extent in both Ncf1-deficient mice and wild type (WT) mice, however, the OCL formation and pre-osteoclast populations were differentially affected in the two genotypes.

## Methods

### Animal procedures

The regional ethical review board in Gothenburg approved this study. The point mutation in *Ncf1* [12] has been backcrossed onto the B10Q background (WT) [6] and kept in breeding. WT mice and mice derived from the same background but with a homozygous mutation in the Ncf1-gene (Ncf1*/*) were housed in a temperature-controlled room with a 06.00–18.00 h light cycle and consumed a soy-free diet (R70, Lantmännen, Stockholm, Sweden) and tap water ad libitum.

### Ovariectomy and treatment

At 7 to 10 weeks of age, female mice were ovariectomized or sham-operated, under isoflurane (Baxter Medical Ab, Kista, Sweden) anesthetization by inhalation. Carprofen (Orion Pharma AB, Animal Health, Sollentuna, Sweden) was administered subcutaneously as postoperative analgesic.

Mice were implanted with slow release pellets containing placebo or 17β-Estradiol (E2) at the time of ovx (0.05 mg E2/kg/day; 90-day release, Innovative Research of America, Sarasota, FL). The animals were randomly divided into 3 groups per genotype (6–9 mice per group): sham + placebo, ovx + placebo or ovx + E2. Treatment continued until study termination (4 weeks).

### Tissue collection

Mice were terminated 4 weeks after ovx or sham operations. At the time of termination, mice were anesthetized with ketamine (Pfizer AB, Täby, Sweden) and medetomidine (Orion Pharma AB), bled and killed by cervical dislocation. Mice body weights were recorded (WT sham 25 ± 3.2 g, WT Ovx 25 ± 3.0 g, Ncf1*/* Sham 25 ± 1.7 g, Ncf1*/* Ovx 25 ± 1.5 g), uteri were weighed and one femur was put in 4 % paraformaldehyde (PFA) for measuring BMD. One femur and two tibiae (for osteoclast differentiation) and one humerus (for FACS analysis) were put in phosphate buffered saline (PBS).

### Osteoclast differentiation

Bone-marrow derived macrophages (BMM) were cultured and stimulated with macrophage colony stimulating factor (M-CSF) and RANKL to induce osteoclast differentiation [13]. Bone marrow was flushed with minimum essential medium Eagle’s alpha modification (αMEM; Gibco, Grand Island, NY, USA) supplemented with 10 % heat-inactivated fetal bovine serum (FBS; Sigma-Aldrich®, St Louis, USA). Cells were incubated for 2 days at 37 °C in a humidified CO_2_ chamber on a culture dish (Corning®, Hickory, NC, USA) with αMEM-medium containing 30 ng/ml M-CSF (R&D system Europe LTD, Abingdon, UK). After two days, the adhering cells (BMMs) were detached, after which 5000 cells in 5 μl αMEM/10 % FBS were seeded at the center of 96-well plates and left to adhere for 10 min. Subsequently, 200 μl medium was added containing either 30 ng/ml of M-CSF (controls), or 30 ng/ml M-CSF + 2 ng/ml of RANKL. After 4 days, the cells were fixed and stained for tartrate-resistant acid phosphatase (TRAP). TRAP-positive cells with ≥3 nuclei were considered osteoclasts. The amounts of TRAP-positive multinucleated osteoclasts were calculated by standardized counting of the midsection of a 96-well by Osteomeasure 3.2.1.0 software (Osteometrics, Inc., Decatur, GA, USA) [14]. The total OCL covered area in the well was calculated by tracing the OCL boundaries. Area per individual OCL was calculated by dividing OCL covered area with number of OCL. Three mice per group were used and assayed in quadruplicate.

### Assessing BMD

After being kept for 2 days in 4 % PFA and subsequently stored in 70 % ethanol, femurs were scanned using peripheral quantitative computed tomography (pQCT) with Stratec pQCT XCT Research M software (version 5.4B; Norland, Fort Atkinson, WI, USA), at a resolution of 70 μm, as described previously [15]. Cortical thickness was determined by one mid-diaphyseal scan at a distance from the distal growth plate corresponding to 36 % of total femur length. This mid-diaphyseal region of mouse femur only contains cortical bone. Trabecular BMD was determined by one metaphyseal scan at a distance from the distal growth plate corresponding to 3 % of the femur length. The trabecular bone region was set as 45 % of the inner total cross-sectional area.

### Single cell preparation and immunophenotyping by flow cytometry

Single cell suspensions for flow cytometry were prepared from bone marrow as previously described [16]. Erythrocytes were lysed using Tris-buffered 0.83 % NH_4_Cl. Cells were then stained with fluorochrome-conjugated antibodies to detect different cell populations (Table 1). Fluorescence-minus-one stained samples were used as controls. Data was acquired on a BD FACS Canto II and analyzed using Flow Jo 8.8.6 (Three Star Inc, Ashland, USA). Macrophages were defined as CD11b^+^ M-CSFR^+^ cells; and pre-osteoclasts defined as CD11b^+^M-CSFR^+^RANK^+^ cells. Median Fluorescence Intensity, MFI, was calculated of RANK expression on CD11b^+^M-CSFR^+^ cells.

**Table 1:** Antibody table

Peptide/protein target	Name of Antibody	Manufacturer, catalog #, and/or name of individual providing the antibody	Species raised in; monoclonal or polyclonal	Dilution used
M-CSFR	APC anti-mouse CD115 (CSF-1R)	Biolegend, #135510	Rat, monoclonal	1:10
CD11b	V450 anti-mouse CD11b	BD Biosciences, #560455	Rat, monoclonal	1:50
CD8	PE anti-mouse CD8	BD Biosciences, #553033	Rat, monoclonal	1:50
CD8	PerCp anti-mouse CD8	BD Biosciences, #553036	Rat, monoclonal	1:50
CD4	V450 anti-mouse CD4	BD Biosciences, #560468	Rat, monoclonal	1:50
CD69	PE anti-mouse CD69	BD Biosciences, #561932	Armenian Hamster, monoclonal	1:30
CD11c	APC anti-mouse CD11c	BD Biosciences, #550261	Armenian Hamster, monoclonal	1:50
MHC II	PerCp anti-mouse I - A/I-E (MHC II)	Biolegend, #107624	Rat, monoclonal	1:50
CD80	Pacific Blue anti-mouse CD80	Biolegend, #104724	Armenian Hamster, polyclonal	1:10
RANK	PE anti-mouse CD265 (RANK)	Biolegend, #119806	Rat, monoclonal	1:20

### Estimation of ROS production

Dihydrorhodamine (DHR) 123 dye was used as a free radical sensor (final concentration of 0.25 μg/ml, Life Technologies, Carlsbad, CA, USA), which forms the fluorescent product rhodamine 123 after oxidation. To quantify the respiratory burst activity, bone marrow cells were incubated at 37 °C for 15 min with phorbol myristate acetate (PMA, 0.5 μg/ml; Sigma-Aldrich). Cells were washed with cold PBS, acquired by a BD FACS Canto II (BD Biosciences, San Jose, CA, USA), and analyzed using Flow Jo software (FlowJo, LLC, Ashland, OR, USA).

### Statistical analysis

Statistical evaluations were performed using IBM SPSS Statistics for Macintosh, Version 22.0.0.0, released 2013 (IBM Corp, Armonk, NY, USA). As bone mineral density measurements are sensitive to sibling controls, statistical analysis was only used to compare mice of the same genotypes. Student’s *t*-test was used for comparison of two groups. Whenever Levene’s test revealed unequal variances between the groups, Welch’s *t*-test was used instead. Logarithmic transformations were used when appropriate to ensure normal distribution of data. *P* values <0.05 were considered statistically significant. GraphPad Prism version 6.0c was used for graphical representations. Reported values are mean ± SD unless otherwise stated.

## Results

### Osteoclastogenesis increases after ovx in WT mice but not in Ncf1*/*-mice

**Fig. 1:**
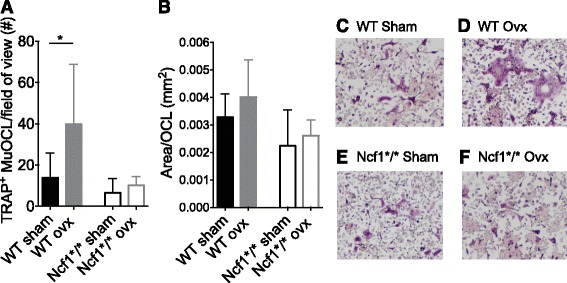
Ovx of WT mice induces osteoclast formation, but not ovx of Ncf1^*/*^-mice. Number **a** and area **b** of OCL formed *ex vivo* in wild-type (WT) and Ncf1-deficient (Ncf1^*/*^) mice after ovx or sham operations. In C-F, representative images of OCL are shown from the respective groups. Three mice per group were used and assayed in quadruplicate. Data displays mean ± SD, *p < 0.05 in sham versus ovx mice

Osteoclastogenesis was induced from bone marrow cells. We found an increase in the number of OCL formed *ex vivo* after ovx in WT mice (Fig. 1a). However, in the Ncf1*/*-mice, the number of OCL formed did not increase after ovx (Fig. 1a). The total OCL area did not differ between the groups (Fig. 1b). We confirmed the reduced estrogen levels after ovx in both genotypes by measuring uteri weights (WT sham 77 ± 41 mg, WT ovx 12 ± 1.6 mg, *p < 0.0001*; Ncf1*/*- Sham 95 ± 30 mg, Ncf1*/*- ovx 13 ± 4.1 mg, *p < 0.0001*). We further confirmed that monocyte/macrophages from WT mice produced more ROS than Ncf1*/*-mice (Geometric mean fluorescence intensity (gMFI): WT sham 542 ± 70, Ncf1*/* Sham 283 ± 89).

### Ncf1 does not regulate ovx-induced bone loss

**Fig. 2:**
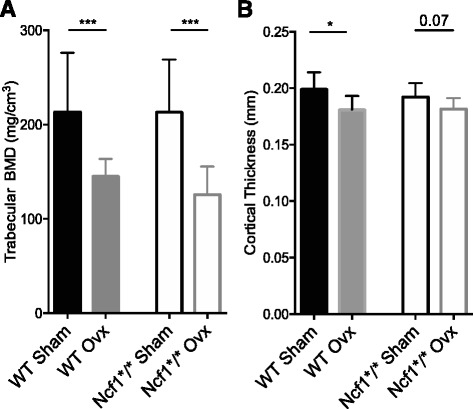
BMD decreases in both WT and Ncf1^*/*^-mice after ovx. Mice were either ovx- or sham-operated for 4 weeks. Trabecular bone mineral density and cortical thickness were evaluated by pQCT **a**-**b**. Data displays mean ± SD, *p < 0.05, ***p < 0.001 in sham versus ovx mice

We measured BMD 4 weeks after ovx in both WT and Ncf1*/*-mice. pQCT measurements revealed equally reduced trabecular BMD after ovx in WT and Ncf1*/*-mice (Fig. 2a). Cortical thickness had decreased significantly in the WT mice after ovx, but only borderline in the Ncf1*/*-mice (Fig. 2b). To investigate a possible rate-dependency of bone degradation after ovx, we also analyzed BMD 2 weeks after ovx in both genotypes. Trabecular BMD had decreased significantly in both genotypes, whereas cortical thickness had not decreased in either genotype at this earlier time point *(data not shown).* We further investigated whether both genotypes responded to E2 treatment after ovx-induced bone degradation. We found that after 4 weeks of treatment, trabecular BMD (WT-E2 692 ± 73 mg/cm^3^, Ncf1*/*-E2 625 ± 116 mg/cm^3^) and cortical thickness (WT-E2 0.23 ± 0.01 mm, Ncf1*/*-E2 0.23 ± 0.03 mm) increased in both WT and Ncf1*/*-mice with this dose of E2. We also confirmed uteri growth after E2 treatment in both WT and Ncf1*/*-mice (WT-E2 208 ± 46 mg, Ncf1*/*- E2 185 ± 60 mg).

### RANK-expressing pre-OCL increase after ovx in Ncf1*/*-mice

**Fig. 3:**
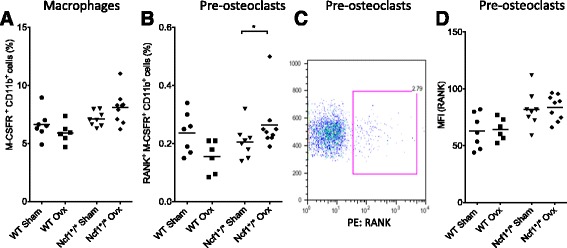
Pre-osteoclasts expand in Ncf1^*/*^-mice after ovx. Bone marrow cells were evaluated using FACS. Populations of macrophages (% CD11b^+^ M-CSFR^+^ cells; **a**, or pre-osteoclasts (% CD11b^+^M-CFSR^+^ RANK^+^ cells; **b** were evaluated after ovx in WT or Ncf1^*/*^ mice. A representative FACS-plot of RANK expression on CD11b^+^M-CSFR^+^ cells is displayed in **c**, and Median Fluorescent Intensity, MFI expression for RANK on CD11b^+^M-CFSR^+^ cells is presented in **d**. Data displays mean ± SD, *p < 0.05 in sham versus ovx mice

The two factors M-CSF and RANKL are important in the formation of OCL [17]. The cytokine M-CSF influences hematopoietic stem cells in the bone marrow to differentiate into the family of macrophages. OCLs forms from this M-CSF-dependent precursor, shared with macrophages. When OCL precursors are incubated with RANKL, these cells can further differentiate through the RANK receptor and fuse into multinucleated OCL. To evaluate the importance of Ncf1 in the formation of macrophages and pre-osteoclasts, we analyzed cells from the bone marrow using flow cytometry. The frequency of macrophages expressing the M-CSF receptor did not differ in either genotype (Fig. 3a). Neither did the frequency of cells expressing F4/80 (results not shown). However, the population of pre-osteoclastic macrophages expressing RANK expanded in Ncf1*/*-mice after ovx, but not in WT mice (Fig. 3b). There was no difference in MFI expression of RANK (Fig. 3d).

### Ovx leads to lymphocyte activation in both genotypes

According to previous studies, lymphocytes are involved in the activation of OCL after ovx [7]. Ovx triggers APCs to activate T-cells in order to stimulate OCL activity. Neither the general dendritic cell (DC) population (CD11c^+^ cells) nor the MHC class II^+^DCs were affected in either genotype 2 or 4 weeks after ovx (Table 2). The CD80^+^DCs expanded in the Ncf1*/*-mice 4 weeks after ovx (Table 2). The general CD4^+^ T-cell population decreased after ovx (Table 2), whereas the CD69^+^CD4^+^ cells increased in both genotypes 2 weeks after ovx (Table 2). Similar results were found for the CD69^+^CD8^+^ cell population (Table 2).

**Table 2:** Lymphocyte frequencies (mean %) after ovx in WT and Ncf1-deficient mice (*n* = 6–9 mice per group, *t*-test comparing ovx vs. sham for each genotype)

Leukocyte population	Sub population	WT Sham (%, ±SD)	WT ovx (%, ±SD)	*p*-value	Ncf1*/* Sham (%, ±SD)	Ncf1*/* ovx (%, ±SD)	*p*-value
4 weeks after ovx							
DCs (CD11c^+^)		2.5 ± 0.9	2.4 ± 0.2	n.s.	2.3 ± 0.7	2.6 ± 0.5	n.s.
	MHC II^+^	44 ± 10	44 ± 9.2	n.s.	49 ± 16	51 ± 16	n.s.
	CD80^+^	19 ± 3.8	17 ± 4.3	n.s	18 ± 4.1	25 ± 8.4	0.04
T-cell (CD4^+^)		1.6 ± 0.3	0.8 ± 0.2	<0.0001	1.2 ± 0.3	0.93 ± 0.3	0.04
	CD69^+^	22 ± 6	26 ± 5.5	n.s	24 ± 8.8	33 ± 8.0	n.s
T cell (CD8^+^)		1.4 ± 0.2	0.74 ± 0.2	<0.0001	0.94 ± 0.4	0.8 ± 0.3	n.s
	CD69^+^	17 ± 7.4	23 ± 3.7	n.s	21 ± 7.7	25 ± 8.0	n.s
2 weeks after ovx							
DCs (CD11c^+^)		7.3 ± 0.9	6.6 ± 0.1	n.s.	6.6 ± 1.0	6.2 ± 1.1	n.s.
	MHC II^+^	40 ± 6.0	38 ± 2.9	n.s.	37 ± 4.8	36 ± 4.4	n.s.
	CD80^+^	30 ± 3.0	24 ± 3.2	0.001	31 ± 4.2	25 ± 4.6	0.03
T-cell (CD4^+^)		2.1 ± 0.5	0.7 ± 0.2	<0.0001	2.3 ± 0.6	1.1 ± 0.5	0.001
	CD69^+^	21 ± 5.3	33 ± 8.4	0.002	19 ± 6.5	26 ± 3.2	0.02
T cell (CD8^+^)		2.2 ± 0.9	0.8 ± 0.2	<0.001	2.0 ± 1.3	0.56 ± 0.16	0.01
	CD69^+^	9.8 ± 2.3	19 ± 5.0	<0.001	12 ± 3.7	25 ± 8.5	0.002

## Discussion

Bone resorption and bone formation shifts with advancing age, leading to loss of bone mass and strength. In postmenopausal women, both age and loss of estrogens contribute to activating osteoclasts, inflammatory cells, and induce ROS production in the bone marrow. In our study, we found that ovx increases OCL formation *ex vivo* in WT mice, accompanied by reduced cortical thickness and trabecular BMD. In Ncf1*/* mice, no increase in OCL formation was seen after ovx, indicating that the Ncf1*/* should retain bone density after ovx. Surprisingly, the Ncf1*/* mice loose BMD similar to WT mice after ovx.

We investigated several possible mechanisms for this found discrepancy. First, we investigated whether the difference was present already on pre-OCL in the bone marrow (defined as CD11b^+^M-CSFR^+^RANK^+^ macrophages) destined to become OCL upon stimulation. The crucial role of Ncf1 for macrophage activation was previously shown where Ncf1 deleted specifically in macrophages regulated chronic inflammation [18–20]. In our study, Ncf1*/* mice showed an expanded population of pre-osteoclastic macrophages expressing RANK after ovx (Fig. 3b), indicating that they are target cells also in this model. One compensatory mechanism could be to increase OCL sensitivity and upregulate RANK. We could however not find any differences in the intensity of RANK expression using FACS (Fig. 3d). In addition, Ncf1*/* did not affect OCL area. This suggests that the OCL retain their efficiency regardless of numbers, and implies that it is the total bone area covered by the OCL that is important. Previous studies have implicated that NOX 1, 2 and 4 are important in bone loss. The OCL has been shown to switch NOX-complex in order to produce the necessary levels of ROS, possibly as a compensatory mechanism to ensure an adequate bone resorption process [21]. NOX 4 was shown to be more active in bone resorption in young mice [22], whereas the NOX 2 complex was shown to be important for bone loss in elderly mice (more than 2 years of age [23]. Those studies verified the importance of the two NOX complexes for bone resorption at different ages, and suggest that NOX 4 may compensate for lost ROS production in Ncf1*/* mice.

The bone remodeling process was comparably regulated by estrogen treatment in this study, as treating ovx mice with E2 resulted in similar increases in cortical thickness and trabecular BMD in both Ncf1*/* and WT-mice. However, interactions between E2 and Ncf1 were possibly concealed in this study due to the high dose and duration of the estrogen treatment.

In previous studies of postmenopausal bone loss, activated T cells and APCs were shown to regulate the bone resorption process [7]. Since Ncf1 regulates auto-reactive T cells through APCs in autoimmunity, we further investigated activated T cells and DCs. We found similar changes at 2 weeks in both WT and Ncf1*/* mice, whereas at 4 weeks after ovx, the CD80^+^DCs population was expanded in the Ncf1*/*-mice. This indicates a possible rate difference, and that a target mechanism lies further up in the hierarchy, such as on the pre-OCL or macropages, or on soluble factors secreted from the activated APCs. TNFα is produced in abundance after ovx, and can induce osteoclastogenesis by itself. A possible discrepancy between OCL formation after stimulation with RANKL, TNFα and other cytokines in the Ncf1*/*-mice will be subject for further studies. Both the increased RANK- expression on macrophages and lingering increased CD80^+^DCs in Ncf1*/*-mice could contribute to an altered bone degradation process, such as we discerned of the cortical bone in Ncf1*/*-mice. The lack of statistically significant difference in cortical thickness of Ncf1*/*-mice could either be due to a too small number of animals used in each group, or it is possible that other more sensitive BMD measurement techniques such as microCT could detect finer details and differences in the rate dependent bone degradation processes, thus revealing additional aspects regarding effects of Ncf1 on bone. The model we used in this study is a global Ncf1-deficiency model, reducing Ncf1 function in all cells. Therefore, possible mechanisms regulated by Ncf1 includes effects on osteoblasts, altered regulation of the cross-talk between osteoblasts and OCL, compensatory ROS production from another NOX or regulating another mechanism altogether, all of which are subjects for future studies.

## Conclusion

Here, using a model of postmenopausal bone loss, we report that Ncf1, an essential subunit of the ROS-producing NOX 2 complex, contributes to OCL formation possibly by regulating pre-OCL. The NOX complexes are active in many physiological and pathophysiological processes. Increasing our understanding of their function will help in producing new targets for alleviating disease.

## Abbreviations

APC: Antigen presenting cell
BMD: Bone mineral density
BMM: Bone-marrow derived macrophages
DC: Dendritic cell
E2: 17β-Estradiol
FBS: Fetal bovine serum
gMFI: Geometric mean fluorescence intensity
M-CSF: Macrophage colony stimulating factor
MFI: Median fluorescence intensity
NADPH: Nicotinamide adenine dinucleotide phosphate
Ncf1: Neutrophil cytosolic factor 1
NOX: NADPH oxidase
OCL: Osteoclast
OVX: Ovariectomy
PBS: Phosphate buffered saline
PFA: Paraformaldehyde
PMA: Phorbol myristate acetate
pQCT: peripheral quantitative computed tomography
RANKL: Receptor activator of nuclear factor κ B ligand
ROS: Reactive oxygen species
TNFα: Tumor necrosis factor alpha
TRAP: Tartrate-resistant acid phosphatase
WT: Wild type
αMEM: Minimum essential medium Eagle’s alpha modification.
